# The burden of colorectal cancer attributable to dietary risk in Middle East and North African from 1990 to 2019

**DOI:** 10.1038/s41598-023-47647-y

**Published:** 2023-11-20

**Authors:** Yahya Pasdar, Fatemeh Khosravi Shadmani, Hawal Lateef Fateh, Davood Soleimani, Behrooz Hamzeh, Mojtaba Ghalandari, Behrooz Moloudpour, Mitra Darbandi

**Affiliations:** 1 Research Center for Environmental Determinants of Health (RCEDH), Health Institute, Kermanshah University of Medical Sciences, Kermanshah, Iran; 2 Cardiovascular Research Center, Kermanshah University of Medical Sciences, Kermanshah, Iran; 3 Nursing Department, Kalar Technical College, Garmian Polytechnic University, Kalar, Iraq; 4 Student Research Committee, Tabriz University of Medical Sciences, Tabriz, Iran

**Keywords:** Risk factors, Cancer, Cancer epidemiology, Cancer prevention

## Abstract

The incidence of colorectal cancer (CRC) is increasing in low- and middle-income countries. This study aimed to estimate the burden of CRC attributable to nutritional risk in the Middle East and North Africa (MENA) region. The GBD 2019 methods were used to estimate age-standardized mortality rates (ASMRs) and disability-adjusted life-years (DALYs) in 2019 and over the past three decades. We evaluated the 30-year trend in DALYs and mortality rates from nutrition-related risks of CRC, from 1990 to 2019 by sex and age groups in 21 countries in the MENA region. The rate of DALYs/100,000 due to diet-related risks for CRC in 2019 was 79.71 (95% UI: 56.79, 98.44) and 65.16 (95% UI: 45.86, 80.95) in men and women, respectively. The percent changes of DALYs/100,000 in men and women were 8.15% and 2.50%, respectively, between 1990 and 2019. The percent changes in ASMRs in men and women were 8.32% and 3.44%, respectively. The highest DALYs and ASMRs were observed in both sexes in the age group 75–79 years and above. The highest percent changes in DALYs/100,000 and ASMRs were observed between 1990 and 2019 in Afghanistan, Egypt, Iran, Iraq, Lebanon, Libya, Morocco, Palestine, Qatar, Saudi Arabia, Sudan and Yemen. DALYs and ASMRs attributed to dietary risk for CRC increased in 21 countries in the MENA region from 1990 to 2019. A modified diet with more fiber, dairy products and less red meat intake is a highly recommended strategy for prevention CRC.

## Introduction

Colorectal cancer (CRC) ranks third in incidence and second in mortality rate in worldwide^1^. The incidence of CRC was estimated at 1.8 million new cases and 881,000 deaths in 2018, and is projected to increase by about 60% by 2030^2^. In low- and middle-income countries, CRC has shown an increasing trend^2^. Age-standardized incidence, mortality rates and disability-adjusted life-years (DALYs) for CRC in the Middle East and North Africa (MENA) are 13.9, 9.8 and 218.7 per 100,000, respectively^3^.

While the exact cause of CRC is still unknown. However, evidence suggests that the incidence of CRC is influenced by genetic, environmental, obesity, behavioral habits (smoking and inactivity) and metabolic risks^4, 5^. The modifiable environmental and behavioral risk factors such as diet and nutritional habits are potentially preventable^6, 7^. Epidemiological studies have demonstrated an association between dietary intake and the risk of CRC. In 2017, a low- fiber diet contributed to 25,561 CRC deaths, with population attributable fractions (PAFs) of 13.7% in China^8^. A study by Mint et al. has shown an association between meat consumption and CRC risk in the MENA region^9^. The association between dairy consumption and risk of CRC in the MENA region in a systematic review indicates conflicting results^10^. In general, diets high in fiber, whole grains, milk and calcium can reduce the risk of CRC^8, 11, 12^, whereas red and processed meats, animal fats, and high-cholesterol foods increase the risk^11^. The results of a meta-analysis (2016) show that "healthy" dietary pattern may reduce the risk of CRC, while "western-style" and "alcohol-consumption" patterns may increase the risk of CRC^13^. A study by Yujiao Deng et al. (2021) showed that CRC deaths and DALYs due to nutritional risks increased by 50.88% and 47.63% respectively, over the past 30 years^6^.

According to the Global Burden of Disease (GBD) study (2015), the attributable burden due to nutritional risks is estimated to be 12.2% and 9% of total DALYs for men and women worldwide in 2015, respectively, making dietary risk a greater burden than smoking or alcohol consumption^14^. This highlights the importance of improving nutrition at the global, regional, and national levels. In fact, diet is one of the modifiable risk factors that can reduce the burden of CRC. Identifying CRC risk factors helps policy makers to design prevention programs. This study aimed to estimate the mortality and burden of CRC attributable to nutritional risks in the MENA region from 1990 to 2019.

## Methods

### Data source, case definition and exposure

This study was conducted using the Global Burden of Disease-2019 (GBD-2019) study, which can be found on the Institute for Health Metrics and Evaluation (IHME) website. Data are presented by country, age, and sex from 1990 to 2019 (https://vizhub.healthdata.org/gbd-compare/). The study population includes the MENA region, which includes 21 countries. Therefore, we extracted data for 21 countries, including Afghanistan, Algeria, Bahrain, Egypt, Iraq, Iran, Morocco, Oman, Palestine, Jordan, Kuwait, Lebanon, Libya Qatar, Saudi Arabia, Sudan, Syria, Tunisia, Turkey, the United Arab Emirates, and Yemen.

The CRC is defined as colon and rectal cancer. International Classification of Diseases-10 (ICD-10) codes were used to represent CRC (C18–C21, D01.0–D01.2, and D12–D12.8)^15^.

Diet-related risks were considered as risk factors. According to GBD study, six dietary risk factors have been evaluated, including diets high in red meat, low in fiber, low in calcium, low in milk, and low in whole grains^6^. Diets low in whole grains consist of consumption of less than 140 to 160 gram/day of whole grains (germ, endosperm and bran in normal proportions) from pancakes, cookies, bread, breakfast cereals, biscuits, tortillas, rice, pasta and others source. Low-milk diets are the average daily consumption of less than 360 to 500 gram/day of milk (high-fat, low-fat and fat-free), excluding soy milk and other plant derivatives. Diets high in red meat include consumption of red meat (gram/day), such as lamb, pork, beef, and goat, but exclude eggs, fish, poultry, and all processed meats. Diets high in processed meat represent any consumption of meat (gr/day) that is preserved by cooking, smoking, salting, or adding chemical preservatives. Diets low in fiber indicate an average intake of less than 21 to 22 gr/day of fiber per day from all sources, especially grains, fruits, legumes, vegetables, and legumes. Low-calcium diets are defined based on an average intake of less than 1.06–1.1 gram/day of calcium from all sources, including cheese, milk, and yogurt^6^.

### Statistical analysis

DALY was computed as follows: DALY = Years lost due to disability (YLD) + years of life lost (YLL). DALYs and age-standardized mortality rates (ASMRs) attributable to dietary risks were presented by country and sex. The age composition of the population in MENA region is different and it is necessary to adjust the effect of age. Therefore, age-standardized estimates are reported. We report age-standardized estimates and 95% confidence intervals (CIs) for rates or numbers of DALYs, deaths, and 10-year percent change from 1990 to 2019 in the MENA region and 21 countries. All rates were per 100,000 persons. Percent change was calculated at three time points by sex and country. All analyses were performed using R software version 4.0.2 (2020.06.22).

### Ethics approval and consent to participate

The study was approved by the ethics committee of Kermanshah University of Medical Sciences (KUMS.REC.1402.056). All methods were carried out by relevant guidelines and regulations.

## Results

The rate of DALYs/100,000 attributed to dietary risk for CRC and the percent change from 1990 to 2019 in MENA countries are shown in Table 1. The rate of DALYs/100,000 attributed to dietary risk for CRC in 1990, 2000, 2010 and 2019 were 70.77 (95% UI 51.69, 92.56), 65.37 (95% UI 48.41, 78.76), 73.70 (95% UI 53.21, 88.64) and 79.71 (95% UI 56.79, 98.44), respectively, among men in the MENA region. The rate of DALYs/100,000 attributed to dietary risk for CRC in 1990, 2000, 2010, and 2019 was 63.59 (95% UI 46.04, 81.94), 59.16 (95% UI 43.98, 70.80), 63.57 (95% UI 44.88, 76.26), and 65.16 (95% UI 45.86, 80.95), respectively, among women in the MENA region.

**Table 1:** Age-standardized DALY rate (per 100,000 population) with 95% uncertainty interval (lower, upper) of colorectal cancer attributable to dietary risk in the Middle East and North Africa from 1990 to 2019.

Country	Sex	DALY (per 100,000 population)				Percent change (%)		
		1990	2000	2010	2019	1990–2000	2000–2010	2010–2019
Afghanistan	Women	89.74 (44.93, 156.97)	84.06 (45.58, 136.79)	90.07 (50.31, 139.66)	96.52 (54.41, 144.96)	− 6.33	7.15	7.15
	Men	77.78 (42.68, 148.17)	72.53 (43.65, 157.84)	77.61 (50.11, 151.75)	82.2 (53.8, 144.27)	− 6.74	7.00	5.92
Algeria	Women	55.53 (39.05, 72.47)	53.54 (36.05, 69.55)	50.07 (31.88, 65.49)	49.4 (31.92, 67.51)	− 3.58	− 6.48	− 1.33
	Men	57.6 (40.91, 75.05)	56.63 (39, 75.4)	52.85 (35.28, 69.85)	53.11 (34.09, 72.61)	− 1.67	− 6.67	0.49
Bahrain	Women	71.35 (46.49, 93.58)	69.31 (45, 89.67)	63.42 (39.55, 83.75)	56.86 (34.54, 78.5)	− 2.85	− 8.49	− 10.35
	Men	97.86 (66.52, 128.75)	85.98 (58.06, 110.86)	87.08 (58.32, 112.65)	73.89 (46.06, 102.52)	− 12.13	1.27	− 15.14
Egypt	Women	46.24 (35.61, 55.15)	43.6 (31.81, 55.09)	48.32 (32.46, 63.98)	51.27 (31.33, 75.65)	− 5.72	10.83	6.10
	Men	51.2 (40.02, 60.99)	49.18 (36.17, 61.58)	51.94 (36.94, 67.19)	60.83 (37.67, 89.12)	− 3.93	5.61	17.10
Iran	Women	59.6 (43.32, 78.61)	55.2 (42.03, 66.38)	61.23 (46.27, 72.14)	65.64 (48.69, 78.1)	− 7.38	10.92	7.20
	Men	71.18 (52.63, 91.36)	64.8 (50.81, 76.58)	75.96 (57.75, 88.32)	87.09 (66.27, 102.71)	− 8.96	17.22	14.65
Iraq	Women	60.83 (39.9, 90.01)	57.9 (41.58, 77.4)	63.08 (45.96, 83.67)	67.36 (47.83, 89.39)	− 4.80	8.94	6.77
	Men	71.94 (44.46, 105.09)	70.64 (51.21, 94.54)	78.32 (56.85, 101.19)	86.89 (62.14, 113.62)	− 1.81	10.88	10.93
Jordan	Women	116.42 (82.96, 156.02)	110.27 (82.02, 139.56)	96 (68.85, 121.04)	91.88 (64.11, 122.94)	− 5.27	− 12.94	− 4.28
	Men	110.76 (79.45, 146.95)	105.09 (76.85, 133.81)	116.83 (83.89, 142.94)	114.02 (79.31, 153.15)	− 5.11	11.16	− 2.40
Kuwait	Women	54.63 (34.89, 73.1)	55.18 (35.35, 71.08)	55.72 (34.43, 73.02)	41.21 (25.4, 57.53)	1.00	0.96	− 26.04
	Men	41.82 (28.09, 53.53)	50.28 (33.33, 64.41)	54.84 (35.86, 71.21)	65.41 (41.01, 90.57)	20.20	9.06	19.28
Lebanon	Women	90.46 (58.35, 126.63)	91.82 (59.68, 121.94)	102.63 (66.84, 140.79)	102.34 (63.49, 146.87)	1.50	11.76	− 0.27
	Men	92.56 (59.5, 128.12)	83.93 (55.55, 109.81)	121.3 (80.22, 158.03)	129.84 (81.43, 182.56)	− 9.32	44.52	7.04
Libya	Women	103.95 (61.94, 161.38)	100.73 (68.39, 132.44)	103.72 (66.07, 137.04)	102.91 (63.52, 150.65)	− 3.10	2.97	− 0.77
	Men	88.53 (48.67, 136.46)	94.81 (67.06, 124.91)	93.79 (63.74, 120.8)	98.36 (60.12, 143.84)	7.09	− 1.07	4.86
Morocco	Women	61.59 (45.08, 77.71)	63.07 (47.07, 78.75)	67.25 (46.44, 89.78)	68.8 (47.61, 96.17)	2.40	6.62	2.30
	Men	58.03 (41.91, 74.59)	58.85 (40.78, 76.22)	60.01 (39.87, 8237)	72.78 (46.45, 101.39)	1.41	1.96	21.28
Oman	Women	60.77 (35.62, 88.84)	61.02 (38.97, 82.42)	66.79 (43.1, 88.05)	56.99 (34.34, 80.24)	0.39	9.46	− 14.67
	Men	55.61 (33.14, 82.5)	61.59 (41.44, 80.42)	68.11 (44.41, 87.44)	55.49 (34.2, 76.59)	10.76	10.58	− 18.52
Palestine	Women	143.26 (93.79, 201.33)	130.64 (99.26, 160.77)	147.97 (118.36, 175.18)	170.43 (133.28, 213.3)	− 8.81	13.27	15.17
	Men	160.67 (99.45, 240.68)	156.23 (123.85, 196.31)	173.9 (141.08, 205.01)	210.81 (164.03, 258.16)	− 2.76	11.31	21.22
Qatar	Women	106.47 (62.87, 152.21)	109.53 (66.15, 149.25)	138.28 (81.61, 183.08)	111.81 (63.7, 152.47)	2.87	26.25	− 19.14
	Men	59.62 (36.37, 87.48)	62.24 (38.08, 86.44)	72.45 (44.29, 99.08)	62.52 (35.92, 89.39)	4.39	16.39	− 13.70
Saudi Arabia	Women	51.04 (29.66, 76.5)	64.73 (42.46, 82.52)	68.6 (44.42, 88.38)	64.97 (39.94, 89.58)	26.82	5.97	− 5.29
	Men	46.97 (28.63, 69.72)	59.88 (41.27, 75.6)	67.39 (46.38, 85.29)	65.93 (41.6, 92.44)	27.48	12.54	− 2.16
Sudan	Women	50.49 (35.33, 67.34)	50.86 (35.71, 69.37)	56.2 (38.02, 78.04)	60.65 (40.78, 87.65)	0.73	10.50	7.90
	Men	51.02 (33.43, 92.47)	53.24 (36.34, 88.64)	61.17 (40.01, 99.72)	69.55 (44.55, 123.75)	4.34	14.89	13.70
Syria	Women	45.24 (30, 61.17)	46.48 (32.56, 59.54)	45 (30.39, 57.88)	45.22 (28.84, 65.94)	2.74	− 3.19	0.50
	Men	47.73 (32.66, 66.18)	51.34 (36.24, 66.79)	48.84 (33.78, 63.77)	52.5 (33.12, 75.55)	7.54	− 4.86	7.50
Tunisia	Women	63.12 (42.74, 82.03)	63.89 (40.99, 84.19)	61.15 (36.56, 85.42)	60.37 (36.57, 87.8)	1.21	− 4.27	− 1.28
	Men	55.34 (38.96, 72.35)	63.21 (43.13, 83.6)	62.79 (38.2, 88)	64.98 (38.8, 94.86)	14.21	− 0.66	3.48
Turkey	Women	81.09 (52.11, 113.3)	64.19 (42.03, 83.34)	67.69 (44.65, 85.98)	64.58 (41.26, 87.88)	− 20.84	5.45	− 4.59
	Men	109.44 (69.74, 153.94)	84.13 (54.39, 109.12)	103.4 (68.84, 129.89)	105.43 (67.79, 140.36)	− 23.13	22.90	1.97
United Arab Emirates	Women	112.04 (60.93, 170)	115.11 (69.47, 157.44)	131.55 (79.6, 182.29)	90.59 (44.86, 158.25)	2.74	14.27	− 31.13
	Men	104.73 (57.69, 160.59)	123.89 (78.68, 171.95)	105.57 (63.75, 145.66)	97.02 (55.86, 142.9)	18.28	− 14.78	− 8.09
Yemen	Women	56.82 (35.1, 86.59)	60.64 (39.76, 89.75)	68.82 (49.78, 93.37)	67.88 (48, 94.19)	6.72	13.49	− 1.36
	Men	57.79 (32.93, 95.6)	61.23 (38.44, 94.48)	70.17 (49.63, 96.64)	70.09 (47.09, 103.67)	5.94	14.60	− 0.10
North Africa and Middle East	Women	63.59 (46.04, 81.94)	59.16 (43.98, 70.80)	63.57 (44.88, 76.26)	65.16 (45.86, 80.95)	− 6.95	7.44	2.50
	Men	70.77 (51.69, 92.56)	65.37 (48.41, 78.76)	73.70 (53.21, 88.64)	79.71 (56.79, 98.44)	− 7.62	12.73	8.15

The rate of DALYs/100,000 attributed to dietary risk for CRC increased among men and women in Afghanistan, Egypt, Iran, Iraq, Lebanon, Libya, Morocco, Palestine, Qatar, Saudi Arabia, Sudan and Yemen from 1990 to 2019. The highest percentage changes in the rate of DALYs/100,000 attributed to dietary risk for CRC between 2010 and 2019 were observed in Egypt, Iran, Iraq, Morocco, and Palestine. In Kuwait, Bahrain, Oman, Qatar, and the United Arab Emirates, the percentage change was negative between 1990 and 2019. In all countries in the MENA region, the rate of DALYs/100,000 attributable to diet-related risk for CRC is higher in men than in women, except in Afghanistan and Qatar.

The ASMRs attributed to diet-related risk for CRC in MENA countries from 1990 to 2019 is shown in Table 2. ASMR attributed to diet-related risk for CRC increased in men and women in Afghanistan, Egypt, Iran, Iraq, Morocco, Palestine, and Sudan from 1990 to 2019. ASMRs attributed to dietary risk for CRC increased by 8.32% in men and 3.44% in women between 2010 and 2019 in MENA countries. ASMRs attributed to diet-related risk for CRC is higher among men than women in MENA countries.

**Table 2:** Age-standardized mortality rates (ASMR) with 95% uncertainty interval (lower, upper) of colorectal cancer attributable to dietary risk in the Middle East and North Africa from 1990 to 2019.

Country	Sex	Age standardized Mortality Rate (Per 100, 000 population)				Percent change (%)		
		1990	2000	2010	2019	1990–2000	2000–2010	2010–2019
Afghanistan	Women	3.41 (2.04, 5.63)	3.17 (1.96, 4.75)	3.48 (2.23, 5.06)	3.78 (2.42, 5.33)	− 6.79	9.54	8.72
	Men	3.21 (1.86, 6.05)	2.99 (1.94, 6.29)	3.26 (2.16, 6.26)	3.5 (2.4, 5.99)	− 7.07	9.11	7.40
Algeria	Women	2.6 (1.85, 3.36)	2.53 (1.76, 3.29)	2.48 (1.61, 3.19)	2.48 (1.6, 3.38)	− 2.70	− 1.98	− 0.12
	Men	2.75 (1.98, 3.52)	2.72 (1.85, 3.64)	2.6 (1.74, 3.37)	2.62 (1.72, 3.59)	− 0.90	− 4.62	0.83
Bahrain	Women	3.17 (2.05, 4.14)	3.12 (2.02, 4.06)	3.22 (2.06, 4.21)	2.85 (1.77, 3.88)	− 1.33	2.96	− 11.42
	Men	4.85 (3.31, 6.24)	4.20 (2.91, 5.40)	4.64 (3.13, 5.99)	3.98 (2.52, 5.44)	− 13.52	10.54	− 14.15
Egypt	Women	1.99 (1.55, 2.37)	1.97 (1.44, 2.51)	2.24 (1.5, 2.97)	2.38 (1.45, 3.55)	− 1.04	13.61	6.04
	Men	1.93 (1.51, 2.29)	1.88 (1.39, 2.35)	2 (1.42, 2.56)	2.32 (1.45, 3.36)	− 2.46	6.19	16.15
Iran	Women	2.63 (1.89, 3.48)	2.50 (1.91, 3.01)	2.83 (2.15, 3.32)	3.09 (2.3, 3.7)	− 4.88	13.15	9.24
	Men	3.14 (2.32, 4.06)	2.90 (2.27, 3.44)	3.44 (2.63, 3.98)	3.93 (2.99, 4.64)	− 7.58	18.49	14.51
Iraq	Women	2.52 (1.66, 3.61)	2.37 (1.75, 3.06)	2.65 (1.97, 3.42)	2.85 (2.1, 3.64)	− 5.85	11.70	7.60
	Men	3.14 (1.96, 4.63)	3.06 (2.26, 4.03)	3.48 (2.59, 4.42)	3.85 (2.78, 4.89)	− 2.61	13.59	10.87
Jordan	Women	4.83 (3.45, 6.44)	4.62 (3.48, 5.79)	4.32 (3.09, 5.46)	4.32 (3.03, 5.71)	− 4.38	− 6.38	− 0.19
	Men	5.04 (3.59, 6.58)	4.76 (3.54, 5.96)	5.54 (4.03, 6.74)	5.36 (3.78, 7.06)	− 5.56	16.47	− 3.36
Kuwait	Women	2.51 (1.64, 3.35)	2.41 (1.57, 3.13)	2.68 (1.73, 3.52)	2.1 (1.28, 2.94)	− 3.95	11.42	− 21.83
	Men	1.95 (1.33, 2.51)	2.40 (1.58, 3.10)	2.7 (1.76, 3.49)	3.3 (2.1, 4.53)	22.97	12.73	22.39
Lebanon	Women	4.1 (2.7, 5.65)	4.29 (2.74, 5.67)	4.94 (3.26, 7.02)	4.83 (3.03, 7.15)	4.80	15.02	− 2.19
	Men	4.47 (2.91, 6.16)	4.23 (2.82, 5.54)	6.07 (4.03, 8.08)	6.32 (4.05, 8.82)	− 5.24	43.52	4.13
Libya	Women	4.18 (2.55, 6.4)	4.05 (2.72, 5.24)	4.27 (2.76, 5.54)	4.23 (2.62, 6.17)	− 2.98	5.27	− 0.77
	Men	3.89 (2.15, 5.93)	4.13 (2.92, 5.38)	4.17 (2.85, 5.33)	4.32 (2.68, 6.21)	6.08	0.90	3.66
Morocco	Women	2.58 (1.89, 3.28)	2.63 (1.97, 3.28)	2.86 (1.97, 3.78)	2.94 (2.02, 3.95)	2.10	8.54	3.00
	Men	2.54 (1.82, 3.28)	2.57 (1.77, 3.32)	2.61 (1.74, 3.56)	3.35 (2.08, 4.68)	1.27	1.33	28.56
Oman	Women	2.78 (1.66, 4)	2.83 (1.83, 3.79)	3.15 (2.02, 4.14)	2.89 (1.78, 4.04)	1.72	11.25	− 8.35
	Men	2.59 (1.56, 3.74)	2.89 (1.96, 3.74)	3.25 (2.09, 4.11)	3 (1.91, 4.04)	11.83	12.37	− 7.78
Palestine	Women	5.86 (3.86, 8.08)	5.53 (4.24, 6.79)	6.37 (5.08, 7.53)	7.48 (5.87, 9.29)	− 5.73	15.18	17.48
	Men	7.29 (4.59, 10.78)	7.25 (5.69, 9.04)	8.36 (6.75, 9.87)	10.2 (7.93, 12.43)	− 0.51	15.26	22.07
Qatar	Women	5.1 (2.93, 7.35)	5.45 (3.27, 7.57)	7.83 (4.61, 10.45)	6.56 (3.69, 9.04)	6.84	43.64	− 16.22
	Men	3.07 (1.88, 4.52)	3.17 (1.94, 4.35)	3.99 (2.45, 5.46)	3.73 (2.18, 5.23)	3.26	25.83	− 6.38
Saudi Arabia	Women	2.29 (1.33, 3.41)	2.93 (1.94, 3.70)	2.98 (1.95, 3.8)	2.81 (1.75, 3.84)	28.19	1.63	− 5.60
	Men	2.14 (1.31, 3.15)	2.81 (1.97, 3.53)	3.11 (2.14, 3.89)	2.92 (1.87, 3.98)	31.10	10.51	− 6.02
Sudan	Women	2.07 (1.49, 2.77)	2.12 (1.49, 2.93)	2.37 (1.66, 3.28)	2.6 (1.78, 3.61)	2.05	12.25	9.61
	Men	2.17 (1.45, 3.87)	2.29 (1.57, 3.76)	2.67 (1.77, 4.29)	3.09 (2.02, 5.45)	5.61	16.32	15.99
Syria	Women	1.97 (1.29, 2.69)	2.19 (1.54, 2.77)	2.27 (1.56, 2.88)	2.23 (1.45, 3.13)	11.24	3.70	− 1.99
	Men	1.94 (1.33, 2.64)	2.16 (1.55, 2.75)	2.23 (1.56, 2.87)	2.34 (1.51, 3.27)	11.55	3.13	4.88
Tunisia	Women	2.91 (1.95, 3.81)	3.00 (1.99, 3.93)	2.83 (1.71, 3.86)	2.82 (1.72, 3.99)	2.88	− 5.49	− 0.53
	Men	2.73 (1.93, 3.53)	3.10 (2.14, 4.04)	3.1 (1.95, 4.23)	3.18 (1.96, 4.61)	13.71	− 0.23	2.62
Turkey	Women	3.48 (2.24, 4.81)	2.77 (1.81, 3.65)	3.23 (2.15, 4.09)	3.15 (2.01, 4.22)	− 20.27	16.30	− 2.41
	Men	4.56 (2.96, 6.31)	3.52 (2.29, 4.59)	4.64 (3.12, 5.83)	4.69 (3.03, 6.21)	− 22.91	31.91	0.98
United Arab Emirates	Women	4.96 (2.62, 7.61)	5.39 (3.24, 7.36)	6.56 (3.95, 8.9)	4.13 (1.94, 7.52)	8.63	21.71	− 37.04
	Men	5.78 (3.16, 8.73)	7.11 (4.51, 9.78)	6.07 (3.75, 8.33)	5.51 (3.29, 7.98)	23.02	− 14.59	− 9.27
Yemen	Women	2.36 (1.45, 3.56)	2.54 (1.68, 3.73)	2.91 (2.13, 3.91)	2.86 (2.01, 4.03)	7.75	14.40	− 1.62
	Men	2.48 (1.41, 4.09)	2.66 (1.71, 4.09)	3.09 (2.25, 4.23)	3.07 (2.12, 4.49)	7.22	16.23	− 0.44
North Africa and Middle East	Women	2.74 (1.99, 3.49)	2.59 (1.89, 3.10)	2.91 (2.06, 3.49)	3.01 (2.13, 3.74)	− 5.74	12.53	3.44
	Men	3.04 (2.24, 3.93)	2.84 (2.13, 3.42)	3.31 (2.42, 3.95)	3.59 (2.55, 4.41)	− 6.47	16.52	8.32

**Figure 1:**
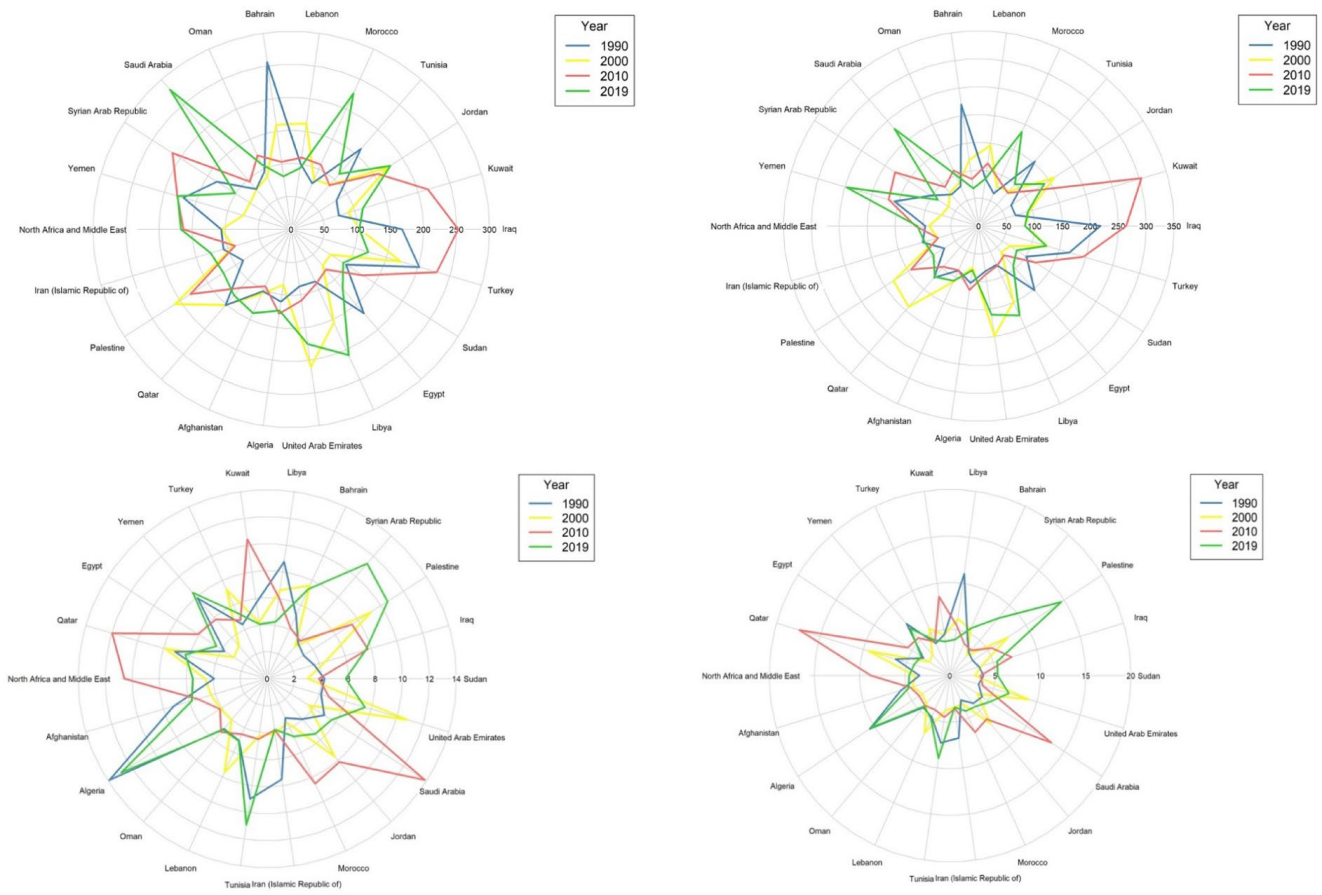
Trend of mortality and burden of colorectal cancer attributed to dietary risk in MENA countries, (**a**) DALYs in men, (**b**) DALYs in Women, (**c**) ASMR in Men, (**d**) ASMR in Women.

Mortality and burden of diet-related risk for CRC in MENA countries for both sexes are shown in Fig. 1. It shows the trend of ASMRs and DALYs attributable to diet-related risks for CRC in MENA countries for both sexes in 1990, 2000, 2010, and 2019. The trend of DALYs/100,000 attributable to nutritional risk increased in Saudi Arabia, Morocco, Jordan, Libya, and Afghanistan for men from 1990 to 2019. The trend of ASMRs attributable to nutritional risk increased in women in Palestine and Tunisia and in men in Yemen, Syrian Arab Republic, Palestine, and Tunisia from 1990 to 2019.

**Figure 2:**
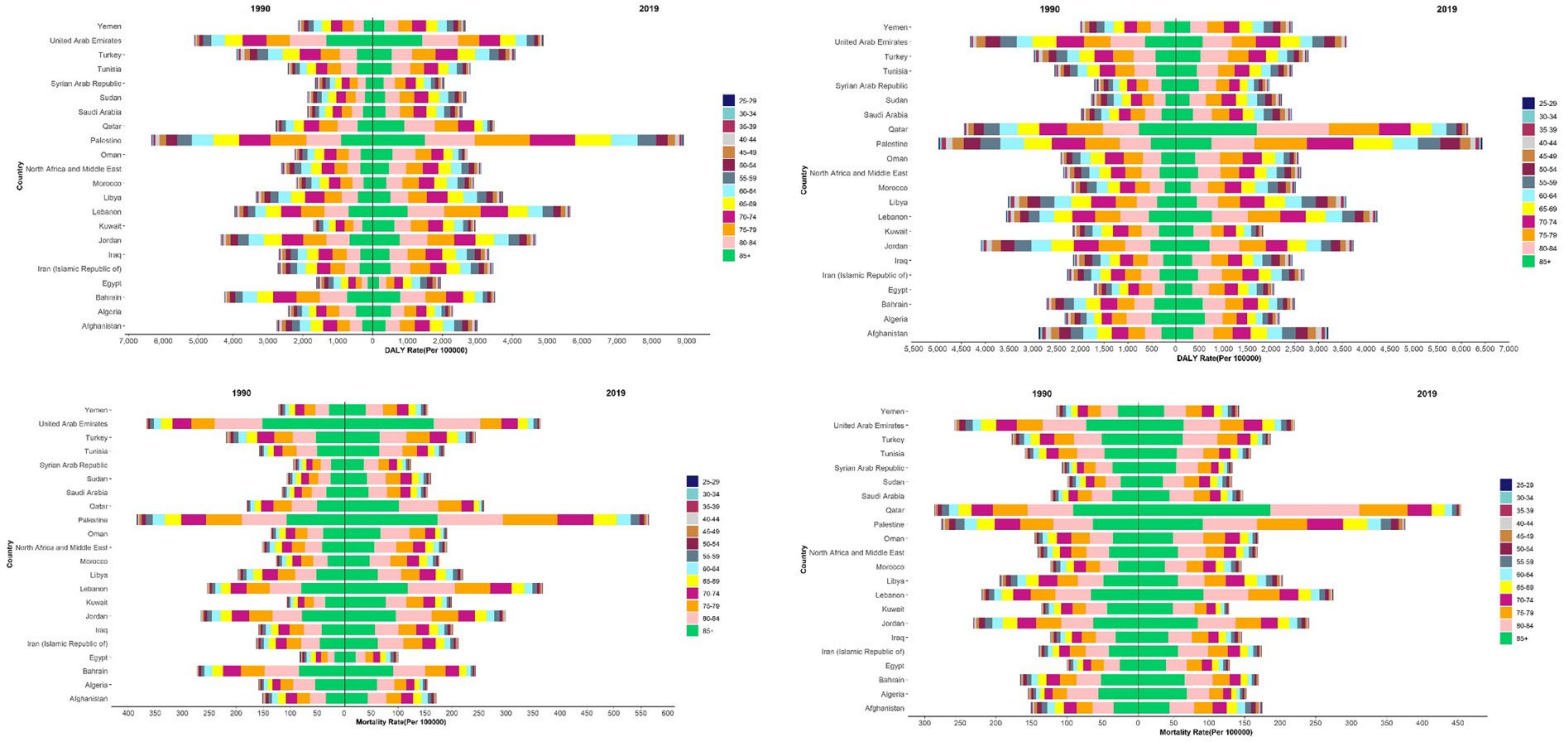
Age trend of mortality and burden of colorectal cancer attributed to dietary risk in MENA countries, (**a**) DALYs in Men, (**b**) DALYs in Women, (**c**) Mortality rate in Men, (**d**) Mortality rate in Women.

Figure 2 DALYs/100,000 and mortality rate attributable to diet-related risks were higher in Palestine, the United Arab Emirates, Jordan, Lebanon, Turkey and Bahrain than in other countries. DALYs/100,000 and mortality rates were higher in the age group 75–79 years and older.

**Figure 3:**
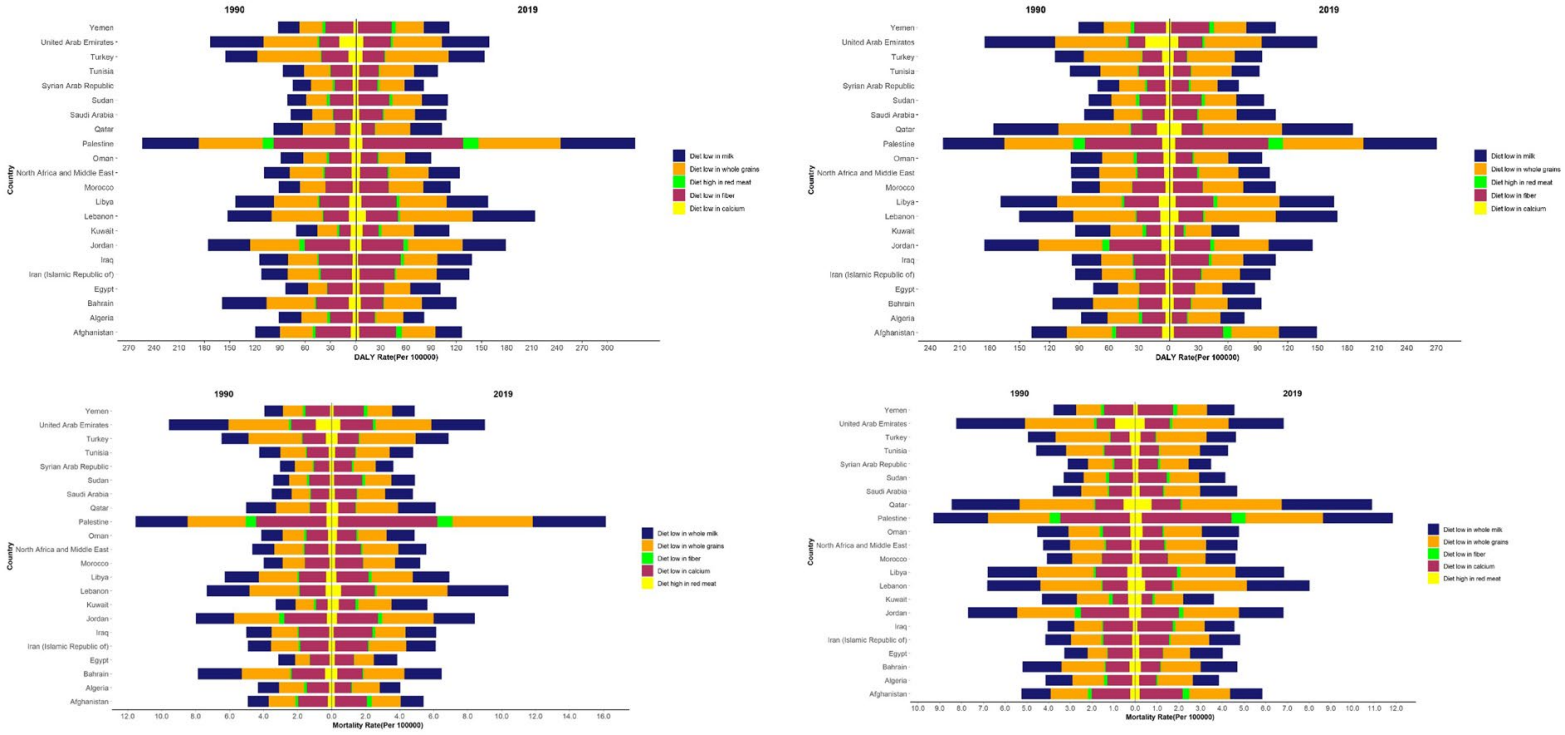
DALYs/100,000 and ASMR of colorectal cancer attributed to diet high in red meat, low in calcium, low in milk, low in fiber and low in whole grain in MENA countries, (**a**) DALYs in Men, (**b**) DALYs in Women, (**c**) ASMR in Men, (**d**) ASMR in Women.

DALYs/100,000 and ASMRs of attributable to diets high in red meat, low in calcium, low in milk, low in fiber and low in whole grains were higher in men in Palestine, the United Arab Emirates, Jordan, Lebanon, Turkey, and Bahrain than in other countries. DALYs/100,000 and ASMRs attributed to a diet high in red meat, low in calcium, low in milk, low in fiber, and low in whole grains for CRC were higher in Palestine, the United Arab Emirates, Jordan, Lebanon, Qatar, Libya, Afghanistan, Turkey, and Bahrain among women than in other countries. A diet low in fiber, milk, and whole grains was associated with a higher risk of CRC than a diet high in red meat and low in calcium in men and women in MENA countries (Fig. 3).

**Figure 4:**
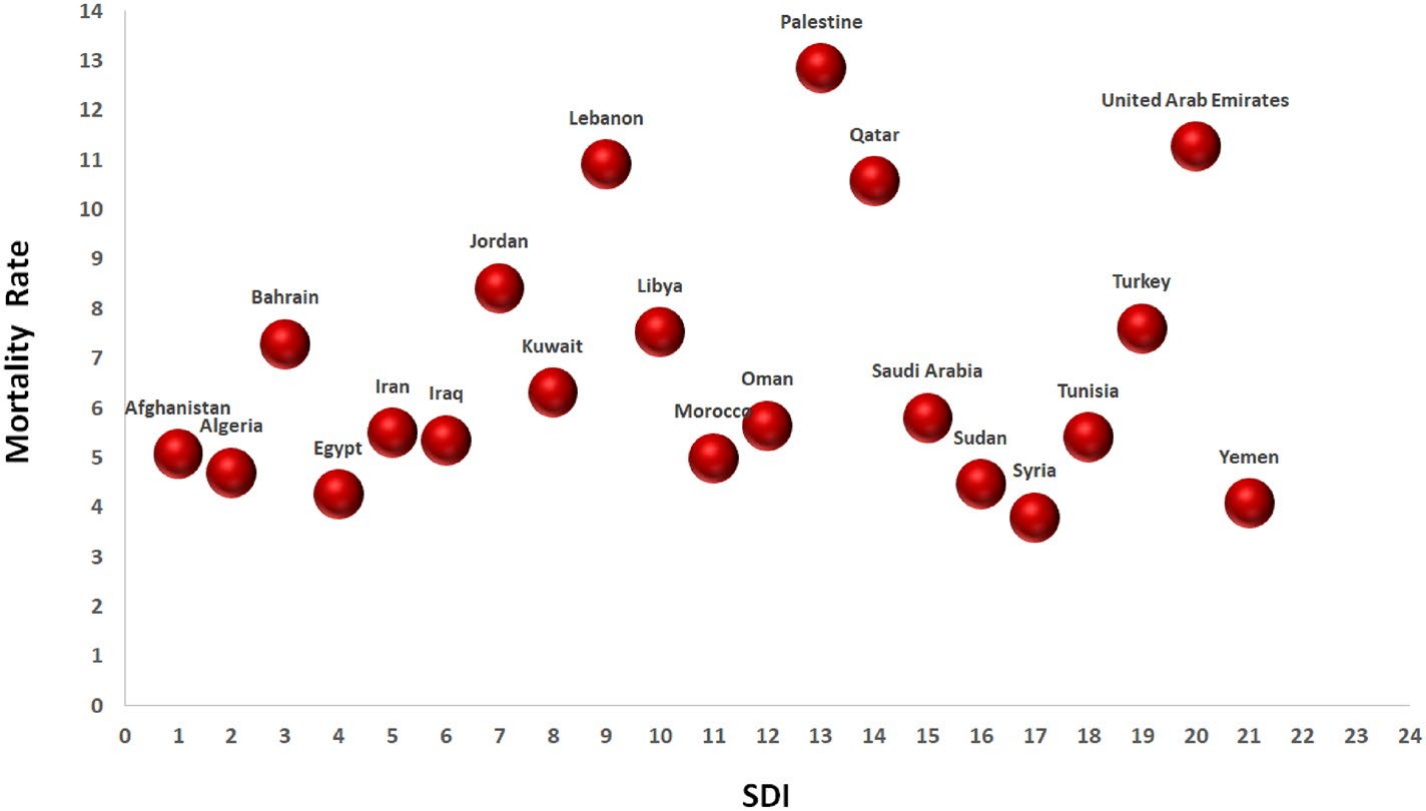
DALYs/100,000 of colorectal cancer attributable to dietary risk in MENA countries by socio-demographic index (SDI) in 2019.

**Figure 5:**
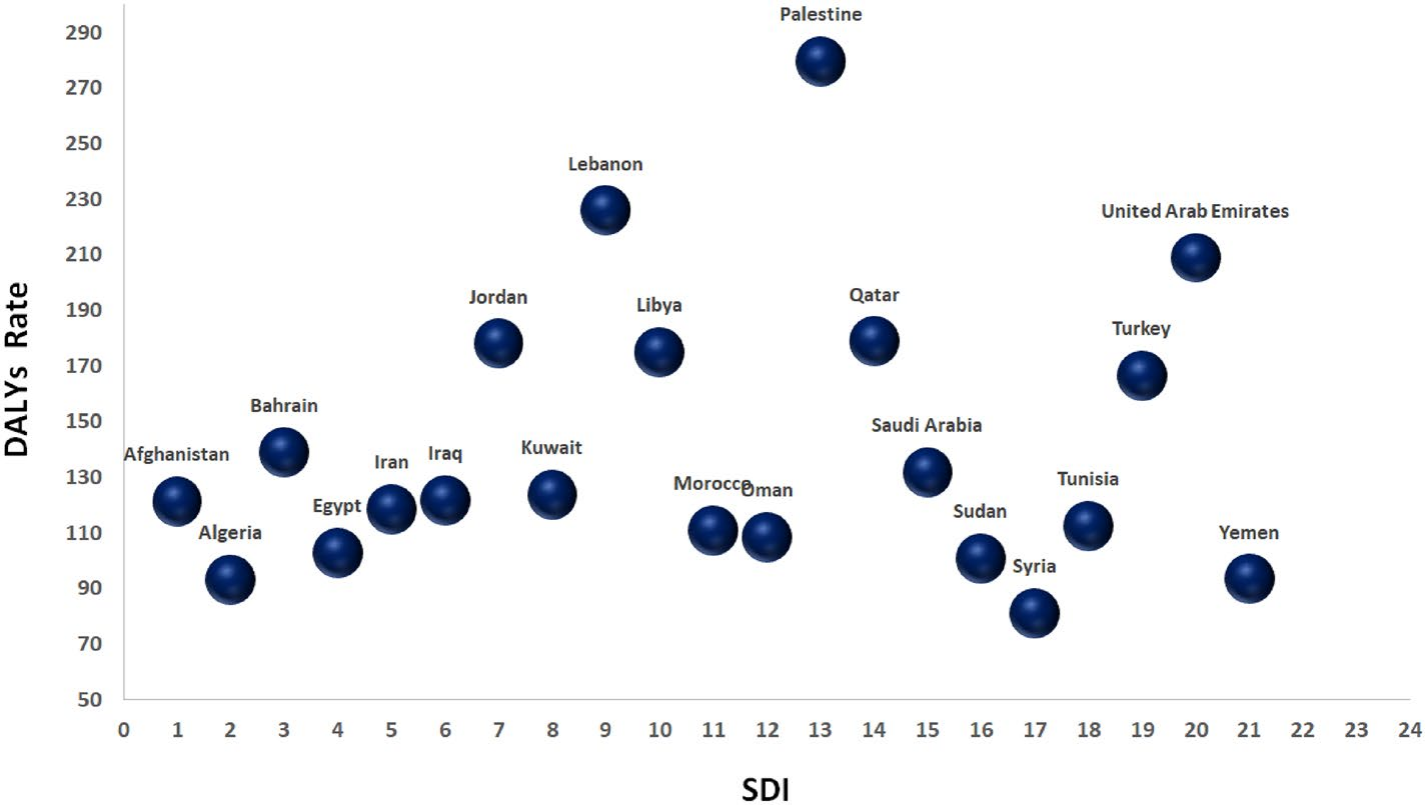
Mortality rate of colorectal cancer attributable to dietary risk in MENA countries by socio-demographic index (SDI) in 2019.

Figures 4 and 5 show that the age-standardized DALYs and mortality rates of CRC due to dietary risk with socio-demographic index (SDI) are not significantly different from each other in MENA countries.

## Discussion

DALYs and ASMRs attributed to nutritional risk for CRC increased in MENA regions from 1990 to 2019. The percent changes related to ASMRs in men and women were 8.32% and 3.44%, respectively. The percent changes related to DALYs/100,000 in men and women were 8.15% and 2.50%, respectively. The highest DALYs and ASMRs were observed in both sexes in the age group 75–79 years and above. The highest percent changes in DALYs/100,000 and ASMRs were observed between 1990 and 2019 in Afghanistan, Egypt, Iran, Iraq, Lebanon, Libya, Morocco, Palestine, Qatar, Saudi Arabia, Sudan and Yemen.

The study by Deng et al. (2019) reported that 32% of deaths and 34% of DALYs worldwide due to CRC were attributable to diet-related factors, and the age-standardized rate (ASR was higher in men than in women^6^. According to the results of a systematic analysis (2022), age standardized incidence and mortality rates were higher in men than in women^3^. The incidence of colorectal cancer has increased in younger age groups (20–49 years) in high-income countries^3, 16^. The increasing trend of diet-related CRC may be attributed to improved screening programs, increased consumption of fast food (high in salt and fat), and increased prices of healthy foods (fruits, vegetables, and dairy products) in some countries.

Our analysis also showed that the lowest DALYs and ASMR of CRC were attributed to diets rich in milk, fiber, whole grains and calcium, and low in red meat in MENA countries. However, the risk of a diet low in fiber, milk, and whole grains was much higher than that of a diet rich in red meat and low in calcium. In general, the role of these four dietary factors (milk, fiber, whole grains, red meat and calcium) was identified as the most important nutritional factors for the incidence of CRC^8, 12, 17–19^. One study showed that a diet low in milk, calcium, and whole grains was responsible for 81.64% of DALYs and 81.61% of deaths for CRC^6^. The results of a systematic review showed that the three most important risk factors for DALYs attributable to CRC in 195 countries, were a low calcium diet (20.5%), alcohol consumption (15.2%), and a low milk diet (14.3%) were for both sexes^20^. A study comparing disease risk factors using GBD data showed that diet-related risk factors increased the global burden more than smoking or alcohol^14^. The results of the study by Vulcan et al. showed that the association between meat consumption and CRC varied by meat type, sex, and tumor location in the intestine^21^. The effects of diet on inflammation and carcinogenesis were examined. In a study by Chou et al., it was reported that a pro-inflammatory diet—characterized by a low intake of fruits and vegetables and a high intake of grains—was strongly associated with an increased risk of CRC. However, the association between pro-inflammatory diet and the risk of CRC may vary depending on the genetic variant of IL-17F, anatomical location and other risk factors^22^. The anti-cancer effect of food has always been an interesting topic for researchers and scientists. Some research has shown that consumption of milk has a protective effect against colon cancer^23, 24^. In addition, vitamin D, calcium, conjugated linoleic acid, butyric acid, and lactose in milk also have certain antitumor effects^23^, and whole grains have anti-cancer properties of fiber, antioxidants, and phytochemicals^25^. This finding highlights the need to improve diet in different ethnicities, regions, and countries. Therefore, diet modification is one of the most important strategies to prevent CRC and can reduce the burden of this disease in worldwide.

Dietary factors increase the risk of CRC through two possible mechanisms, including increasing weight and causing inflammation. Thus, a balanced diet helps to maintain a balanced weight and prevents obesity and its consequences^26–29^. According to scientific evidence, obesity is an important risk factor for CRC^30–32^. Obesity causes inflammation in the body and especially in the intestine. Therefore, inflammation due to obesity is one of the possible factors that increase the risk of various cancers, especially CRC in people, which may lead to CRC due to pro-inflammatory effects and oxidative stress^33, 34^. Therefore, a balanced diet with maintaining a balanced weight and reducing hormones and inflammatory markers such as leptin, adiponectin IL-6, and TNF-α may be a preventive strategy against CRC. Leptin is a risk factor for CRC^35, 36^, and circulating leptin levels are high in obese individuals who have leptin resistance^37^. Epidemiological studies have shown that decreased plasma adiponectin levels are inversely associated with colorectal cancer risk^38, 39^. TNF-α and IL-6 are also secreted from adipose tissue, which is an important inflammatory factor in the acute inflammatory reaction, and is involved in the pathogenesis of CRC^40, 41^.

DALYs/100,000 and ASMRs of attributable to diets high in red meat, low in calcium, low in milk, low in fiber, and low in whole grains were higher in Palestine, the United Arab Emirates, Jordan, Lebanon, Turkey and Bahrain than in other countries. The percentage of DALYs due to CRC attributable to a low calcium and low milk diet in the MENA region was similar to the global percentage (20.6% vs. 20.5%), lower than in African regions (Western, Eastern, Central and Southern Sub-Saharan Africa), and higher than Western Europe, Australasia, Central Europe, Eastern Europe and Central Asia^20^.

Some limitations in this study were unavoidable. Part of the limitation is related to countries’ screening programs and the changes that have occurred in screening programs over the past 30 years. Some countries with poor health systems may not have a proper cancer registration system. The study of a large region of 21 countries is one of the strengths of this study. The results of this study provide data from 21 countries and allow comparability among countries in the region.

## Conclusion

DALYs and ASMRs attributed to diet-related risk for CRC increased in 21 countries in the MENA region from 1990 to 2019, and this increase was greater in men than in women. The highest DALYs and ASMR were observed in the age group 75–79. Afghanistan, Egypt, Iran, Iraq, Lebanon, Libya, Morocco, Palestine, Qatar, Saudi Arabia, Sudan and Yemen had the highest percent changes in DALYs/100,000 and ASMRs between 1990 and 2019. A diets high in milk, fiber, whole grains, and calcium and low in red meat is associated with a lower risk of CRC in MENA countries. Therefore, dietary modification with an increase in fiber and dairy products intake and a decrease in red meat intake is highly recommended strategy for prevention CRC.

## Data Availability

The data sets generated during this study are available from the correspondence author on reasonable request via email.
